# Herpes Zoster Myelitis Mimicking Myelin Oligodendrocyte Glycoprotein (MOG) Antibody Disease: A Case Report

**DOI:** 10.7759/cureus.81782

**Published:** 2025-04-06

**Authors:** Tetsuya Oyama, Kazuya Omichi, Nobuyuki Iwade, Hirotaka Nakanishi

**Affiliations:** 1 Neurology, Yokkaichi Municipal Hospital, Yokkaichi, JPN

**Keywords:** herpes zoster myelitis, mri, myelin oligodendrocyte glycoprotein antibody-associated disease, neuromyelitis optica spectrum disorder, varicella-zoster virus

## Abstract

Identifying the cause of myelopathy is difficult because associated clinical and imaging findings are nonspecific. The onset pattern and magnetic resonance imaging (MRI) findings are important for the diagnosis. Herein, we present the case of a 70-year-old woman hospitalized with acute-onset weakness of the lower limbs. Blood and cerebrospinal fluid tests did not reveal any abnormalities that could have been the cause. Cerebrospinal fluid was negative for varicella-zoster virus (VZV)-DNA. Spinal cord MRI revealed an H-sign in the central gray matter of the conus medullaris, suggesting myelin oligodendrocyte glycoprotein antibody-associated disease (MOGAD)-induced spinal cord inflammation. Intravenous methylprednisolone (IVMP) was initiated; however, the patient’s symptoms did not improve. No anti-MOG antibodies were detected. During hospitalization, shingles appeared on the skin at the same level as the spinal cord lesions. In the repeat cerebrospinal fluid test, VZV-DNA was negative the first time, but later turned positive. We subsequently initiated treatment with acyclovir, and paralysis and bladder-rectum disorders improved. This case study provides important insights for patients with myelopathy. First, it is difficult to distinguish herpes zoster myelopathy from MOGAD because herpes zoster myelopathy presents as an H-shaped lesion in the conical area. Second, when treating myelopathy, virological confirmation via cerebrospinal fluid examination should be repeated until other diseases are diagnosed.

## Introduction

Identifying the cause of myelopathy is difficult because findings and imaging lesions are generally nonspecific [1]. For diagnosis, information such as the onset pattern, course, magnetic resonance imaging (MRI) findings, and additional clinical features must be considered [1]. In MRI examinations for myelopathy, the location, size, shape, and contrast pattern of the lesion are evaluated and are useful for diagnosis [2]. The H-sign of the central gray matter in the conical region is characteristic of myelin oligodendrocyte glycoprotein antibody-associated disease (MOGAD) [3].

Varicella-zoster virus (VZV) infections can trigger neurological complications; however, spinal cord complications are rare [4]. In typical cases, neurological symptoms appear approximately one to two weeks after the appearance of a rash, and these symptoms occur at the spinal cord level corresponding to the rash area. However, some patients do not present with a rash [5]. On MRI, T2 hyperintense lesions are often observed in the spinal cord, whereas long segmental lesions that extend over three or more vertebral bodies are common [6,7]. For diagnosis, it is useful to confirm the presence of the virus using spinal fluid tests. However, the sensitivity of polymerase chain reaction (PCR) testing for VZV DNA is low [8].

Herein, we report a case of herpes zoster myelitis in which the H-sign was observed in the central gray matter of the conus. Initially, we suspected MOGAD and initiated steroid treatment; however, the diagnosis changed when a skin rash appeared on the left buttock during the examination.

## Case presentation

A 70-year-old woman was hospitalized because of weakness in both lower limbs. She had a history of breast cancer, uterine fibroids, and dyslipidemia and was taking pitavastatin calcium hydrate. She had no allergies and did not smoke or consume alcohol. The patient developed weakness in both the lower limbs within several days. Her upper limb strength was normal; however, her lower limb strength decreased to manual muscle testing (MMT) level 2 on both sides. The patellar tendon reflex increased, whereas the Achilles tendon reflex did not. Babinski’s sign was negative. There was loss of sensation in the buttocks and perineum, and the distal part of the ankle joint exhibited dull tactile and thermal sensations. Urinary retention was observed, and the anal sphincter had lost its ability to contract during rectal examination. There was no back pain suggestive of spinal cord infarction and no visual acuity loss, hiccups, or vomiting suggestive of neuromyelitis optica spectrum disorder (NMOSD). Blood tests revealed no obvious abnormalities, and the number of cells in the spinal fluid did not increase. VZV-DNA and VZV-immunoglobulin G (IgG) tests of the cerebrospinal fluid were negative (Table 1).

**Table 1:** Laboratory test results on admission to our hospital CSF: cerebrospinal fluid; PCR: polymerase chain reaction; VZV-IgG: varicella-zoster virus immunoglobulin G

Laboratory Investigation	Patient Value	Reference Range
White blood cell counts (/μL)	5840	3300-8600
Hemoglobin (g/dL)	13.4	11.6-14.8
Platelet count (×10^4^/μL)	19.9	15.8-34.8
Glucose (mg/dL)	105	73-110
C-reactive protein (U/L)	0.27	0-0.14
CSF cell count (/μL)	1	0-5
CSF glucose (mg/dL)	63	50-75
CSF protein (mg/dL)	60	10-40
CSF VZV-IgG	<2	0-4
CSF VZV DNA PCR	Negative	

**Figure 1:**
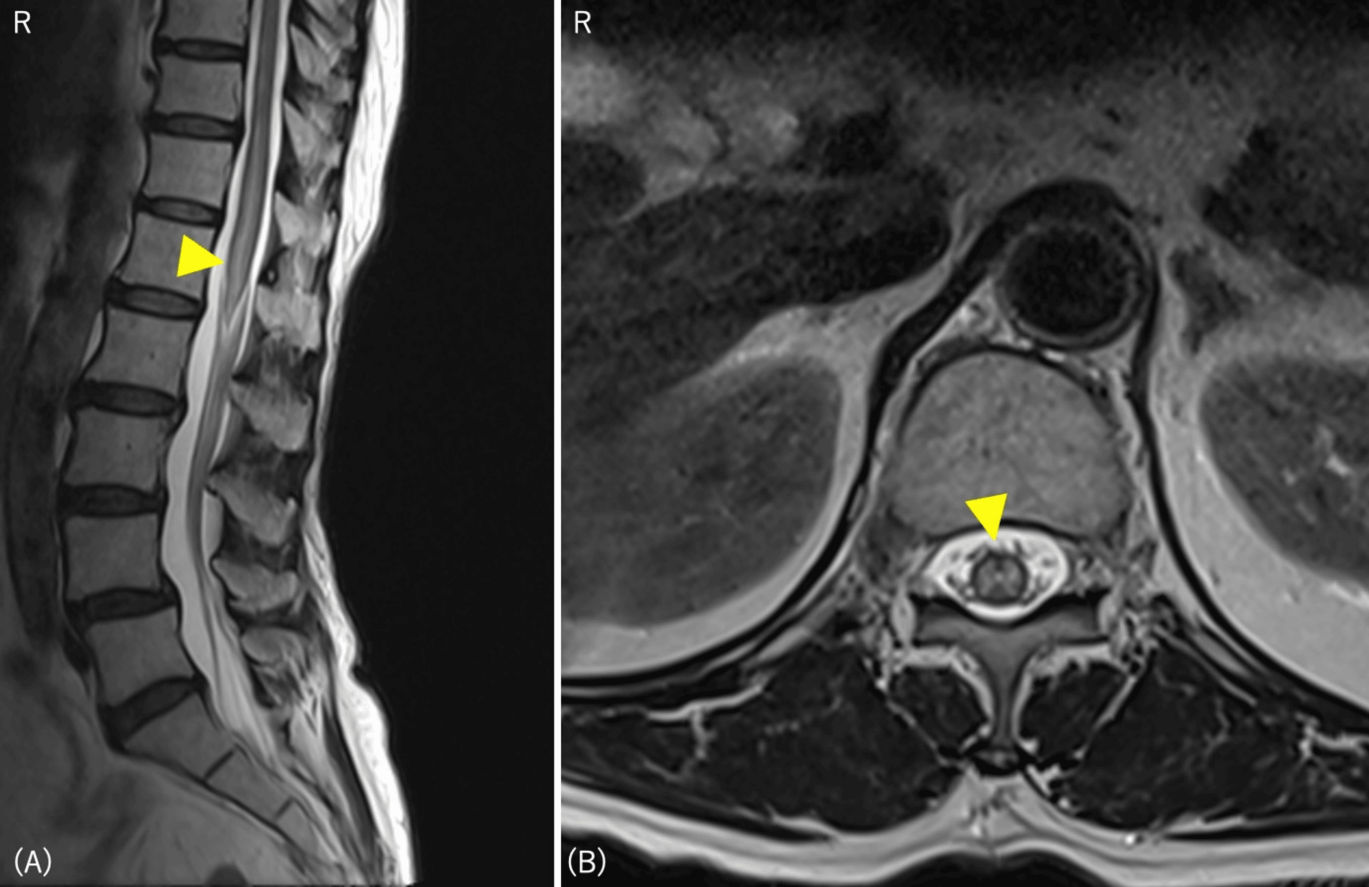
Spinal MRI scan (T2-weighted) on the first day of hospitalization (A) Sagittal section showing intramedullary hyperintensities at the level of the Th12-L1 vertebrae; (B) axial section: H-sign observed in the central gray matter.

Spinal cord MRI revealed intramedullary hyperintensities at the level of the Th12-L1 vertebrae on T2-weighted images. An H-sign was observed in the central gray matter of the axial section, and no contrast effect was observed (Figure 1).

The patient had acute-onset myelopathy, and we suspected inflammatory diseases such as NMOSD and MOGAD. MRI of the spinal cord revealed an H-sign in the central gray matter of the conus medullaris, and we suspected MOGAD. On day two of hospitalization, steroid therapy was initiated. We subsequently administered three courses of intravenous methylprednisolone (1,000 mg of methylprednisolone per day for three days, three weeks in a row), but the patient's lower limb muscle strength remained unchanged at an MMT level of 2. In the cell-based assay (CBA), tests for MOG antibodies in the blood and cerebrospinal fluid and aquaporin-4 (AQP4) antibodies in the blood were all negative (Table 2).

**Table 2:** Results of the investigation for the cause of myelopathy AQP4: aquaporin-4; CBA: cell-based assay; CSF: cerebrospinal fluid; ELISA: enzyme-linked immunosorbent assay: MOG: myelin oligodendrocyte glycoprotein

Laboratory Test	Results
Serum AQP4 (ELISA)	Negative
Serum AQP4 (CBA)	Negative
Serum MOG (CBA)	Negative
CSF MOG (CBA)	Negative

**Figure 2:**
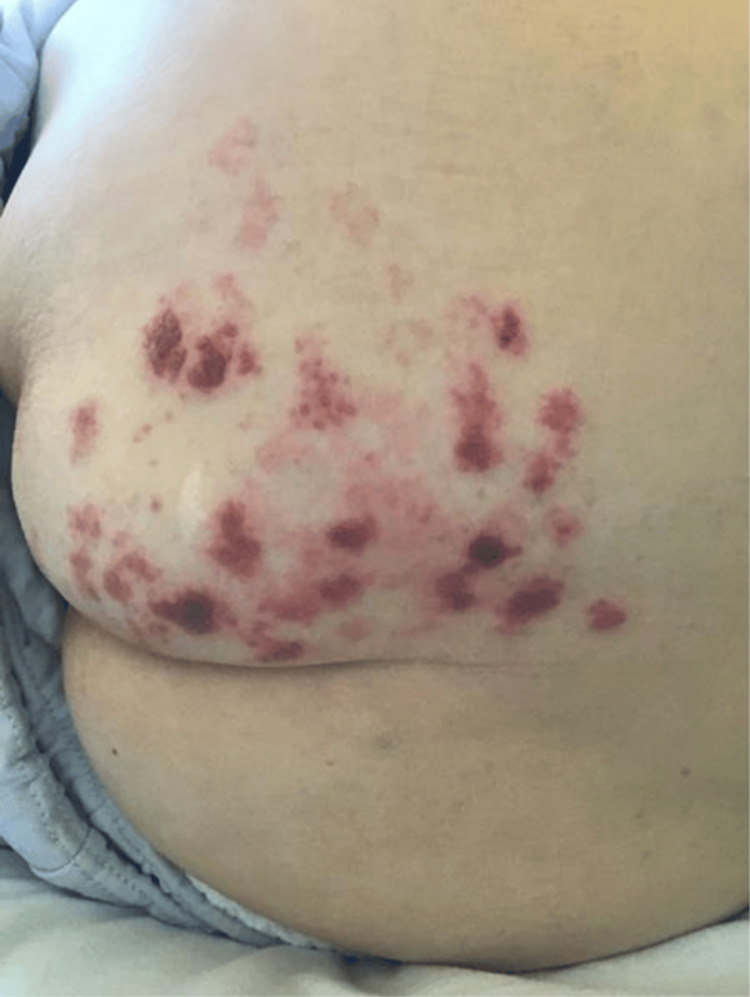
Lateral skin rash on the left buttock A blister rash was observed on the left buttock, and the VZV antigen test was positive.

On the 28th day of hospitalization, a rash was found on the left buttock, and the VZV antigen was detected in the rash (Figure 2).

Reexamination of the spinal fluid revealed a slight increase in the number of cells, whereas VZV DNA was detected in the cerebrospinal fluid (Table 3).

**Table 3:** Laboratory test results on the 28th day of hospitalization CSF: cerebrospinal fluid; VZV DNA PCR: Varicella-zoster virus-DNA polymerase chain reaction

Laboratory Investigation	Patient Value	Reference Range
CSF cell count (/μL)	10	0-5
CSF glucose (mg/dL)	61	50-75
CSF protein (mg/dL)	61	10-40
CSF VZV DNA PCR	Positive	

**Figure 3:**
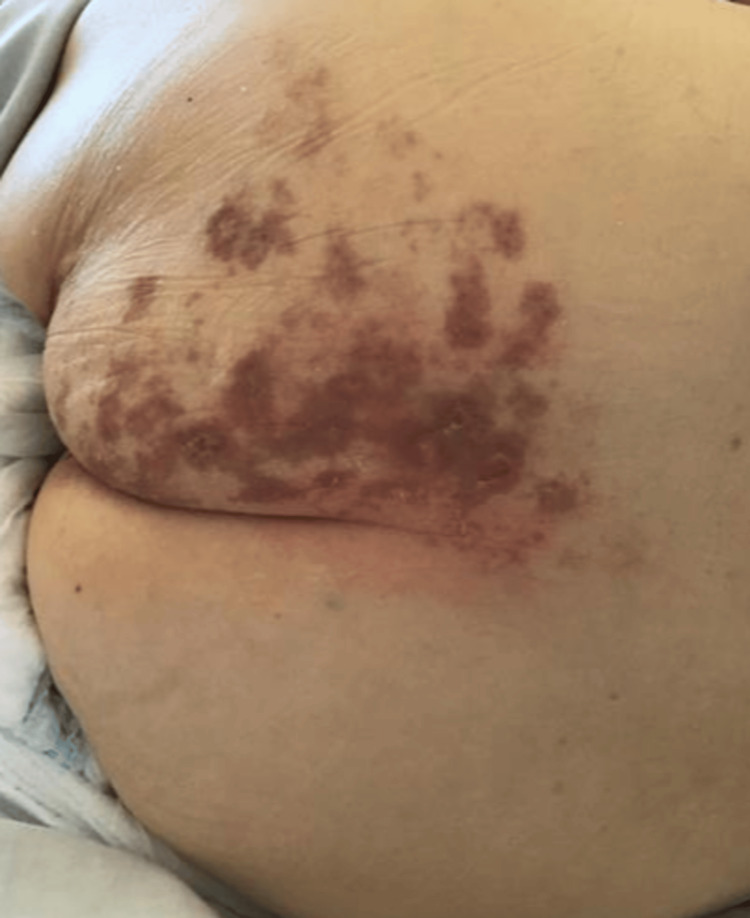
Lateral skin rash on the left buttock after drug treatment A blister rash subsided after drug treatment.

We diagnosed the patient with VZV myelitis and initiated treatment with 250 mg of acyclovir three times a day. After starting the treatment, her lower limb muscle strength improved slightly, and she was able to move against gravity. The patient was also able to urinate and defecate independently. Furthermore, the signal change on the T2-weighted spinal-cord MRI disappeared. We administered acyclovir for 21 days, ending the treatment when VZV-DNA was no longer detected in the cerebrospinal fluid. The skin rash subsided after the drug treatment (Figure 3).

On day 62 of hospitalization, the patient was transferred to another hospital for rehabilitation.

## Discussion

In the present case, we learned the following lessons while searching for myelopathy: first, Herpes zoster myelopathy can cause characteristic imaging changes in MOGAD. Second, when treating myelopathy, virological confirmation using spinal fluid tests should be repeated until other diseases are diagnosed.

In myelopathy, an extremely acute onset indicates a vascular disorder. An acute onset over several days suggests an inflammatory disorder or infection [1]. In this case, the patient developed the disease acutely over several days, and NMOSD, multiple sclerosis (MS), MOGAD, and infectious diseases were considered as differential diagnoses. MOGAD is a demyelinating disease distinct from MS and NMOSD. The most common clinical manifestation is optic neuritis, which can also present as myelitis or cortical encephalitis [9]. Several studies have reported that MOGAD myelitis likely affects the lower thoracic and conus medullaris [10]. Another characteristic is a T2 hyperintensity restricted to the gray matter of the spinal cord, known as the H-sign [3]. The etiology of gray matter T2 hyperintensity in MOGAD myelitis is unclear, but it is thought to reflect demyelination of the gray matter, as oligodendrocytes containing MOG are present in the gray matter of the spinal cord [3]. This patient showed an H-sign in the central gray matter of the conus medullaris, which is a characteristic imaging finding in MOGAD myelitis. Herpes zoster myelitis typically causes neurological symptoms a few days to weeks after the appearance of a rash, and the symptoms occur at the level of the spinal cord corresponding to the rash area [5]. However, more than half of these cases follow an atypical course, and in some cases, there is only a mild or no rash [5]. The patient had no rashes or pain at the spinal level at the disease onset. One month after admission, a rash appeared at the spinal level. Currently, there are no data on the high levels of the spinal cord that are most likely to develop herpes zoster. There have been several reports of conus medullaris myelitis presenting with Elsberg syndrome similar to the present case [11]. Magnetic resonance imaging (MRI) scans of herpes zoster myelitis often show T2 hyperintense lesions in the spinal cord, many of which are long segmental lesions extending over three or more vertebral bodies [6]. The lesion was localized to two vertebrae, Th12-L1. In addition, because there was no rash or pain, we suspected MOGAD based on the H-sign in the axial section.

The diagnosis of herpetic myelitis requires detection of the virus using a spinal fluid test [8]. PCR has high specificity but low sensitivity for the detection of VZV DNA [8]. In our patient, VZV-DNA was negative in the initial cerebrospinal fluid test after admission but was found to be positive in a retest after the rash appeared. Several studies have reported that the timing of the test is a factor in negative results. If the test is performed too early, the amount of virus is too low, whereas if it is performed too late, the virus may clear the system [12]. In the present case, the test result was negative at an early stage of the disease but later became positive, possibly because of steroid treatment. The detection of VZV-IgG antibodies in cerebrospinal fluid is a highly sensitive test [13,14]. However, they may be negative in the early stages of the disease. In the present case, the initial VZV-IgG levels were not elevated. Therefore, it is important to use PCR and antibody tests to increase the sensitivity of spinal fluid tests. It is also important to repeat the test if there are doubts, even if the initial test result is negative.

Few evidence-based treatments exist for herpes zoster myelitis; however, treatment with intravenous acyclovir for 10-14 days and oral corticosteroids for at least one week is generally acceptable [15]. The overall prognosis is good; however, some patients have serious neurological sequelae [5]. The patient was transferred to our hospital and was unable to walk despite the use of assistive devices. If we had been able to diagnose and treat patients earlier, better outcomes may have been achieved.

## Conclusions

In conclusion, we describe a case of herpes zoster myelitis that presented with an H-sign in the conical portion that was difficult to distinguish from MOGAD. It is important to use PCR and antibody tests to diagnose herpes zoster myelitis. However, it may be negative in the early stages of the disease. When treating myelopathy, virological confirmation using spinal fluid examination should be repeated until other diseases are diagnosed.
